# Ecology and spatiotemporal dynamics of sandflies in the Mediterranean Languedoc region (Roquedur area, Gard, France)

**DOI:** 10.1186/s13071-015-1250-2

**Published:** 2015-12-18

**Authors:** Jorian Prudhomme, Nil Rahola, Céline Toty, Cécile Cassan, David Roiz, Baptiste Vergnes, Magali Thierry, Jean-Antoine Rioux, Bulent Alten, Denis Sereno, Anne-Laure Bañuls

**Affiliations:** Centre IRD, UMR MIVEGEC (IRD 224 - CNRS 5290 – Université Montpellier), F34394 Montpellier, France; Faculté de Médecine, Université Montpellier, 2 rue Ecole de médecine, 34000 Montpellier, France; Faculty of Science, Department of Biology, Ecology Section, ESRL Laboratories, Hacettepe University, 0680 Beytepe Ankara, Turkey; UMR INTERTRYP (IRD - CIRAD 177), Centre IRD, F34394 Montpellier, France

**Keywords:** Sandflies, South of France, Ecology, Spatiotemporal dynamics

## Abstract

**Background:**

Phlebotomine sandflies are hematophagous insects widely present in Western Mediterranean countries and known for their role as *Leishmania* vectors*.* During the last ten years, the risk of leishmaniasis re-emergence has increased in France. However, sandfly biology and ecology in the South of France remain poorly known because the last detailed study on their spatiotemporal dynamics was performed over 30 years ago. The aim of the present study was to update our knowledge on sandfly ecology by determining their spatiotemporal dynamics and by investigating the relationship between environmental/climatic factors and the presence and abundance of sandflies in the South of France.

**Methods:**

An entomological survey was carried out during three years (2011–2013) along a 14 kilometer-long transect. The findings were compared with the data collected along the same transect in 1977. Data loggers were placed in each station and programmed to record temperature and relative humidity every six hours between April 2011 and November 2014. Several environmental factors (such as altitude, slope and wall orientation (North, East, West and South)) were characterized at each station.

**Results:**

Four sandfly species were collected: *Phlebotomus ariasi* and *Sergentomyia minuta*, which were predominant, *Ph. perniciosus* and *Ph. mascittii*. Sandfly activity within the studied area started in May and ended in October with peaks in July-August at the optimum average temperature. We found a positive effect of altitude and temperature and a negative effect of relative humidity on *Ph. ariasi* and *Se. minuta* presence. We detected interspecific differences and non-linear effects of these climatic variables on sandfly abundance. Although the environment has considerably changed in 30 years, no significant difference in sandfly dynamics and species diversity was found by comparing the 1977 and 2011–2013 data.

**Conclusion:**

Our study shows that this area maintains a rich sandfly fauna with high *Ph. ariasi* population density during the active season. This represents a risk for *Leishmania* transmission. The analysis revealed that the presence and abundance of *Ph. ariasi* and *Se. minuta* were differently correlated with the environmental and climatic factors. Comparison with the data collected in 1977 highlighted the sandfly population stability, suggesting that they can adapt, in the short and long term, to changing ecosystems.

## Background

Phlebotomine sandflies (Diptera, Psychodidae) are hematophagous insects generally active at dusk and during the night. They are abundant in peri-urban and rural environments, often close to human and domestic animal populations. Although it is thought that sandflies do not fly far away from their breeding sites, one species (*Phlebotomus. ariasi*) can move further than 1 km [1]. Sandflies are present in temperate regions during the summer and in tropical regions throughout the year [2]. They are widely distributed in Western Mediterranean countries [3–5]. In France, excluding oversea territories, five sandfly species have been described so far: *Phlebotomus ariasi* Tonnoir, 1921, *Ph. perniciosus* Newstead, 1911, *Ph. mascittii* Grassi, 1908, *Ph. sergenti* Parrot, 1917, *Ph. papatasi* (Scopoli, 1786) and *Sergentomyia minuta* (Rondani, 1843).

*Phlebotomus* species are known for their role as vectors of medically important pathogens, such as the parasitic protozoa of the genus *Leishmania* (the causative agents of leishmaniasis) [6], the *Bartonella bacilliformis* bacterium [6] and arthropod-borne viruses (Phlebovirus and Vesiculovirus) recognized as human pathogens (Toscana virus, Naples virus and Sicilian virus) [7, 8]. In the Languedoc region, *Ph. ariasi* is the main proven vector of *Leishmania infantum,* while *Ph. perniciosus* is the second one [9, 10].

In the last 10 years, the risk of emergence or re-emergence of Leishmaniasis, according to the National Reference Center of *Leishmania* [11], and Phlebovirus (e.g. Toscana virus in the following papers [12, 13]) has considerably increased in France. On the other hand, the last detailed study on the spatiotemporal dynamics of sandflies in the South of France was carried more than 30 years ago [10]. Climatic variables, especially temperature, are important factors for the distribution of pathogens and their vectors. These relationships must be dissected in the context of the current climate change. For instance, at the meteorological station of Montpellier-Fréjorgues, located about 60 km from our study area, an increase of 1.895 °C (±0.068) in the mean annual temperature between 1946 and 2004 was observed [14]. This study highlights the importance of climate change and the effect of environmental modifications on the dynamics and changes in sandfly populations. Furthermore, the current increase of leishmaniasis distribution in France underlines the necessity to improve our knowledge about the ecology of these insect vectors. Therefore, the objective of this work was to investigate the ecology and spatiotemporal dynamics of sandfly populations and especially of the two main species encountered in the study area: *Ph. ariasi*, the main vector of *L. infantum*, and *Se. minuta*, not involved in *L. infantum* transmission. First, we assessed the effect of climatic and environmental factors on the dynamics of these two species that are characterized by different ecology and life history traits. We then compared our results with those of the study by Rioux et al. [10] to identify modifications in sandfly seasonal dynamics and abundance after 30 years.

## Methods

### Study area

**Fig. 1:**
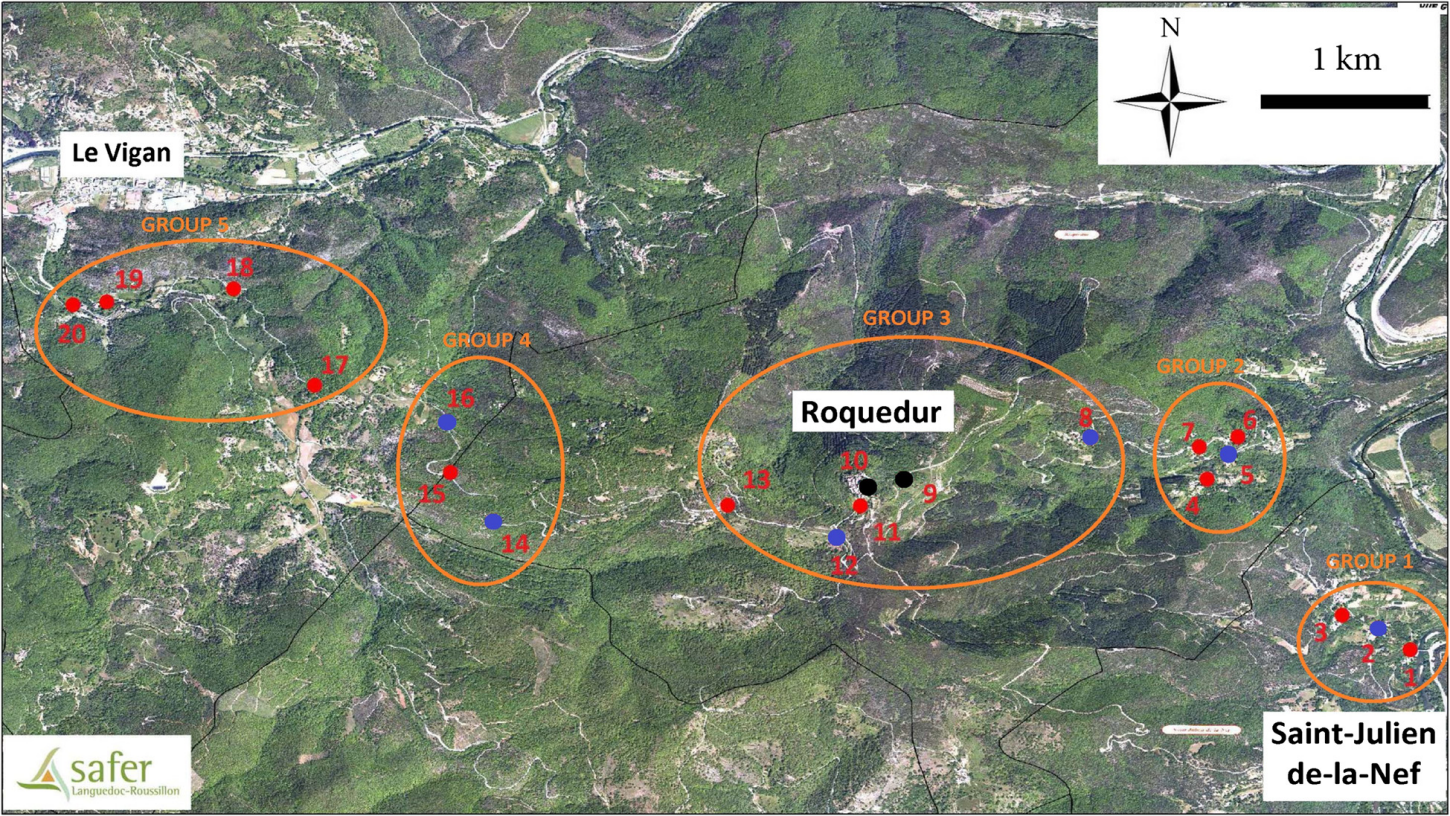
Map of the study area. The twenty stations are represented on the map. The color of each station circle indicates the type of capture: red = sticky and light trap captures; blue = sticky trap captures; black = light trap capture. Groups of altitude defined according to Rioux et al. [10] classification are also indicated: group 1, Hérault valley (100-300 m); group 2, Mid-slope/South side (300-400 m); group 3, Summit (500-600 m); group 4, Mid-slope/North side (300-500 m); and group 5, Arre valley (200-300 m)

The field study was performed in the South of France, on the “massif de l’Oiselette” upland between the “Hérault” (Ganges, Hérault) and “Arre” (Le Vigan, Gard) valleys along a 14 km transect that links the villages of Saint-Julien-de-la-Nef and le Vigan, including Roquedur-le-Haut (at 601 m above sea level) (Fig. 1). This transect is characterized by a succession of rural and semi-rural environments along a road. The weather conditions are those typical of the Mediterranean sub-humid climate [15] and the area is characterized by the presence of “Garrigue” vegetation, such as *Quercus ilex* and *Quercus pubescens*.

The “massif de l’Oiselette” area was chosen because a first spatiotemporal study on sandfly was conducted by Rioux et al. [10] in the same geographical region between May and October 1977. Twenty sampling stations were selected (Table 1) of which 14 were in common with the previous study. These stations were selected according to two criteria: stations already studied by Rioux et al. [10] and stations with favorable environment (wall, houses, animals, etc.) for sandflies regularly distributed along the 14 km transect. This allowed comparing the data on sandfly densities collected at different altitudes on the two opposite sides of the massif in 1977, 2011, 2012 and 2013. Moreover, human and canine leishmaniasis caused by *L. infantum* is endemic in this area. Twenty one autochthonous human visceral leishmaniasis cases were notified in the Gard Department between 1999 and 2012 [16]. Moreover, during the current study, several cases of canine leishmaniasis were observed (unpublished data).

**Table 1:** Sampling stations in the study area (LT: CDC miniature light traps; ST: Sticky Traps)

Station number	Group^b^	Coordinates		Altitude (m)	Biotope	Traps	Wall orientation (degree)
		North	East				
ST01^a^	1	43.96548	3.686828	175	hamlet	LT + ST	117
ST02^a^	1	43.96663	3.685075	228	hamlet	ST	325
ST03^a^	1	43.96733	3.683149	244	hamlet	LT + ST	20
ST04^a^	2	43.97462	3.675871	321	hamlet/hutch	LT + ST	16 and 140
ST05^a^	2	43.97595	3.677038	322	rural	ST	150
ST06	2	43.97687	3.677551	341	kennel	LT + ST	147
ST07^a^	2	43.97632	3.675457	354	hamlet	LT + ST	140
ST08	3	43.97683	3.669611	443	hamlet	ST	177
ST09	3	43.9746	3.659549	586	poultry farm	LT	/
ST10	3	43.97416	3.657616	606	poultry farm	LT	/
ST11^a^	3	43.97317	3.657214	603	hamlet	LT + ST	275
ST12^a^	3	43.97144	3.655944	573	rural	ST	200
ST13^a^	3	43.97321	3.650088	539	rural	LT + ST	170
ST14^a^	4	43.97235	3.637441	417	rural	ST	232
ST15	4	43.97495	3.635153	397	rural	LT + ST	175 and 231
ST16^a^	4	43.97765	3.634949	362	rural	ST	110
ST17^a^	5	43.97961	3.627845	343	rural	LT + ST	178
ST18^a^	5	43.98478	3.623463	282	rural	LT + ST	185
ST19	5	43.98409	3.616661	255	hamlet	LT + ST	240
ST20^a^	5	43.98392	3.614822	245	hamlet/sheep barn	LT + ST	50

^a^Stations present in the study by Rioux et al. [10]

^b^Groups, according to the classification of Rioux et al. [10]: 1, Hérault valley (100-300 m); 2, Mid-slope/South side (300-400 m); 3, Summit (500-600 m); 4, Mid-slope/North side (300-500 m); and 5, Arre valley (200-300 m)

A variety of domestic animals that are potential sandfly hosts (chickens, sheep, ducks, geese, horses, rabbits, cats and dogs) are present in this area. Some sampling stations were located in rural areas because various wild animals could also be sandfly hosts [17].

### Sandfly collection and identification

Sandflies were collected using CDC miniature light traps (LT) (John W. Hock Co. FL, U.S.A.) and sticky traps (ST) (20 × 20 cm sheets of white paper soaked with castor oil) [18] each month, between May and November, in 2011, 2012 and 2013. Nineteen stations were sampled in 2011 and an additional one (station 8) was added in 2012 and 2013 (Table 1).

In 14 sampling sites (Table 1), one or two LT were set up (inside and/or outside houses, animal barns, etc.) and were operated between 18:00 pm and 08:00 am for two nights. Over three years, a total of 329 LT were set up during 649 nights of trapping. In 18 stations (Table 1), a total of 12,196 ST (mean: 230 ST per station) were placed in various biotopes, inside and around human dwellings and animal housing, close to the vegetation and inside crevices in the walls. The ST were collected after two nights.

Captured specimens were transferred individually into 1.5 ml Eppendorf tubes with 90 % ethanol and labeled. Prior to mounting, the sandfly head, genitalia and wings were removed. Wings and body were stored separately for future geometric morphometric and genetic analysis, respectively. The head and genitalia were cleared in Marc-André solution (chloral hydrate/acetic acid) and mounted in chloral gum [2]. Specimen identification was individually verified based on the morphology of the pharynges and/or the male genitalia or female spermathecae, as described by Abonnenc [2], Lewis [3] and Killick-Kendrick et al. [19].

In 1977, 4927 ST, changed every 15 days, were placed in 22 stations between May and October [10]. The environment of the studied area has considerably changed in 30 years. According to the data from the French “Institut National de la Statistique et des Etudes Economique” (INSEE), the human population and the number of houses in this area (Roquedur and Saint-Julien-de-la-Nef) have increased over the years, whereas the numbers of farms has decreased (Table 2). To compare the current and the 1977 ST data, sampling stations were grouped, based on the station altitude (from 100 m to 606 m) and the slope, in five different groups (Table 1), according to the classification used by Rioux et al. [10], 1) Hérault valley (100-300 m), 2) Mid-slope/South side (300-400 m), 3) Summit (500-600 m), 4) Mid-slope/North side (300-500 m) and 5) Arre valley (200-300 m).

**Table 2:** Main demographic data for Saint-Julien-de-la-Nef, Roquedur-le-Haut and Le Vigan in 1982, 1988, 1990, 1999, 2000 and 2009 (Institut National de la Statistique et des Etudes Economiques). Only these years were available in the INSEE web archive

	Population			Number of houses			Number of farms		
Year	Saint Julien de la Nef	Roquedur	Le Vigan	Saint Julien de la Nef	Roquedur	Le Vigan	Saint Julien de la Nef	Roquedur	Le Vigan
1982	99	116	4517	/	/	/	/	/	/
1988	/	/	/	/	/	/	11	11	42
1990	140	133	4523	95	105	2557	/	/	/
1999	119	192	4448	94	112	2645	/	/	/
2000	/	/	/	/	/	/	8	6	12
2009	124	218	3959	126	130	2629	/	/	/

### Collection of climatic data

To investigate whether variations in local temperature and relative humidity could affect sandfly presence or abundance, 20 temperature/humidity data loggers (iButton hygrochron, DS 1923) were placed at each station in areas close to the traps. They were programmed to record temperature (°C) and relative humidity (RH%) every six hours from April 2011 to November 2014. Analyses were performed using the data recorded during each trapping period (three days). The mean/minimum/maximum temperature and the mean/minimum/maximum relative humidity were then computed. At each location, a handheld GPS (Magellan Triton 2000) was used to record the coordinates. Results were transferred to the ArcGIS v9.3 GIS software to produce a map (Fig. 1). Data were collected on the habitat, wall orientation, slope (summit and South or North side) and site characteristics. No climatic data were available for 1977.

### Data analysis

Analyses were performed separately for ST and LT to determine the relation between the presence/abundance of sandflies and different parameters (such as altitude, slope, wall orientation, mean/minimum/maximum temperature and mean/minimum/maximum relative humidity). Sandfly density was calculated as the number of sandflies per m^2^ for ST and as the number of sandflies by trap per night for LT. Data exploration was based on the protocol described by Zuur et al. [20].

The effects of the environmental and climatic variables on the presence of the different sandfly species were analyzed using the Generalized Linear Model (GLM) with binomial distribution. The effects of the environmental and climatic variables on the abundance of the various sandfly species were analyzed using Generalized Additive Models (GAMs) to detect non-linear relationships. As the data showed a significant over-dispersion, the model was fitted using a negative binomial error distribution and a logarithm link [21]. The explanatory variables were: altitude, slope, wall orientation, mean/minimum/maximum temperature and mean/minimum/maximum relative humidity. Model selection was performed using the Akaike information criterion for each variable and a forward stepwise model selection procedure [22]. Validation was performed based on Zuur et al. [23]. Statistical analyses were performed using the R statistical package, version 3.1.2 [24], with the packages mgcv, mass, gam, lattice and MuMin among others.

These analyses were only performed on *Ph. ariasi* and on *Se. minuta*. It was not relevant to include *Ph. perniciosus* and *Ph. mascittii* due to the low number of collected specimens.

The temperature effects on the estimates of inter-annual average sandfly abundance (2011–2013) for *Ph. ariasi* and *Se. minuta* were analyzed using GAMs with negative binomial distribution, as described above. We assumed that a three-year survey was long enough to bring informative results about the relationships between annual climatic variables and annual sandfly abundance (*Ph. ariasi* and *Se. minuta*).

## Results

### Sandfly fauna

Overall, 15,488 specimens (7949 males and 7539 females) belonging to four sandfly species were collected in the 20 stations over three years (Table 3). *Ph. ariasi* (93.23 %) was the predominant species in the study area, whereas *Se. minuta* (6.18 %), *Ph. perniciosus* (0.48 %) and *Ph. mascittii* (0.11 %) were less abundant.

**Table 3:** Number and relative abundance (%) of sandfly species in the study area during the three-year survey (sampling with light and sticky traps). The details of the captures by type of traps are summarized in Table 4

Year	Species	Number^a^	Females	Males	Percentage (%)^b^
2011	*Phlebotomus ariasi*	2866	934	1932	90.52
	*Sergentomyia minuta*	258	109	149	8.15
	*Ph. perniciosus*	39	10	29	1.23
	*Ph. mascittii*	3	3	0	0.09
	Total	3166	1056	2110	100
2012	*Ph. ariasi*	4508	2830	1678	92.83
	*Se. minuta*	320	116	204	6.59
	*Ph. perniciosus*	21	0	21	0.43
	*Ph. mascittii*	7	7	0	0.14
	Total	4856	2953	1903	100
2013	*Ph. ariasi*	7065	3361	3704	94.63
	*Se. minuta*	379	162	217	5.08
	*Ph. perniciosus*	15	1	14	0.20
	*Ph. mascittii*	7	6	1	0.09
	Total	7466	3530	3936	100
	Total	15488	7539	7949	100

^a^Total number of specimens collected by CDC light traps and sticky paper in all sampling stations

^b^Relative abundance: nx/N * 100, nx: number of individuals belonging to species x, N: total number of sampled individuals

During the three years, the distribution of each sandfly species in the different stations did not change significantly (Kruskal-Wallis test, *p-*value = 0.5706, *p-*value = 0.131, *p-*value = 0.815, *p-*value = 0.7715 for *Ph. ariasi*, *Ph. perniciosus*, *Ph. mascittii* and *Se. minuta* captured by ST: and *p-*value = 0.6487, *p-*value = 0.6411, *p-*value = 0.2282, *p-*value = 0.9989 for *Ph. ariasi*, *Ph. perniciosus*, *Ph. mascittii* and *Se. minuta* captured by LT, respectively). *Ph. ariasi* and *Ph. perniciosus* were present in almost all sampling sites, while *Se. minuta* was more abundant in rural biotopes (especially, stations 5, 12, 13, 15 and 17).

### Seasonal dynamics

**Fig. 2:**
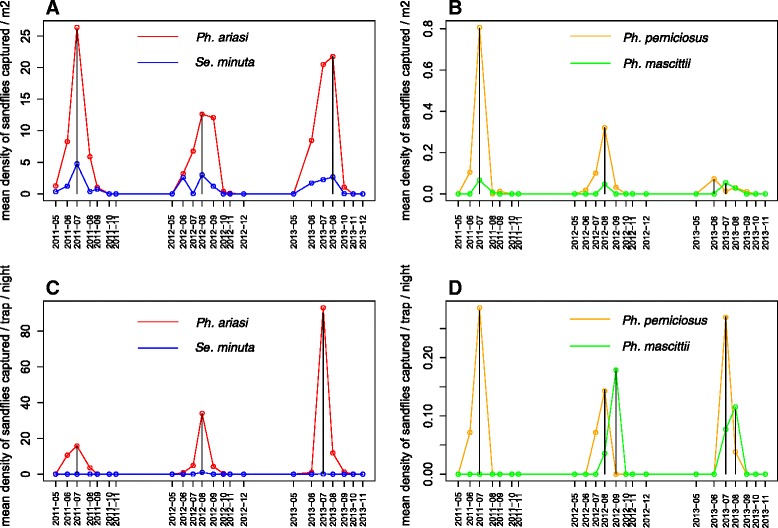
Seasonal dynamics of the sandfly species captured by sticky traps (**a** and **b**) and light traps (**c** and **d**) in the study area. Red line: *Phlebotomus ariasi*; blue line: *Sergentomyia minuta*; orange line: *Ph. perniciosus*; green line: *Ph. mascittii*; black line: maximum peak of density

Overall, *Ph. ariasi* was the predominant species during the active season. *Se. minuta* was also continuously present during the season, although it was less abundant compared with *Ph. ariasi*. Conversely, *Ph. perniciosus* and *Ph. mascittii* were rare throughout the studied period (Fig. 2).

**Fig. 3:**
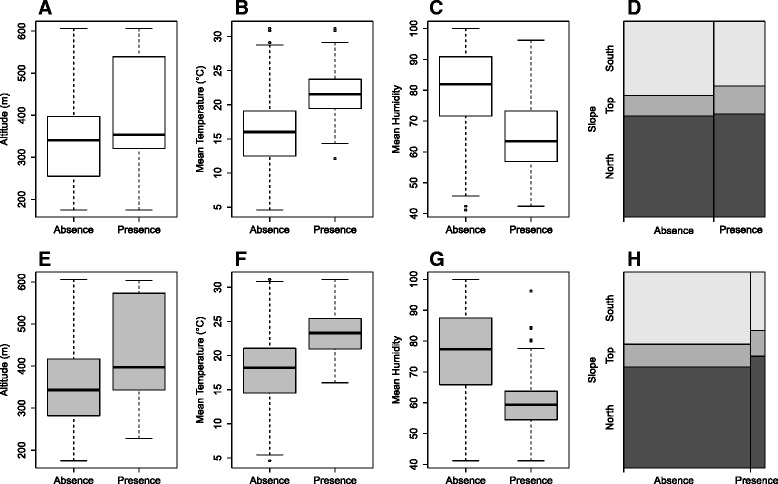
Boxplot showing the number of times *Phlebotomus ariasi* (upper histograms) or *Sergentomyia minuta* sandflies were found (presence) or not (absence) in sticky and light traps according to the altitude (**a** and **e**), relative humidity (**b** and **f**), temperature (**c** and **g**) and slope features (**d** and **h**)

There was a difference in terms of density peaks between trap types. The mean density (based on ST captures) for all sandfly species was highest in July during the first two years. However, in 2013, *Ph. ariasi* and *Se. minuta* were slightly more abundant in August, *Ph. perniciosus* in June and *Ph. mascittii* in July (Fig. 2a and b). Based on the LT capture data, *Ph. ariasi* and *Ph. perniciosus* showed a peak of density in July, while *Ph. mascittii* was more abundant in August (Fig. 2c and d). Nevertheless, the monthly density of the four considered species did not significantly change during the three years of study (Kruskal-Wallis test, *p-*value = 0.9362, *p-*value = 0.8196, *p-*value = 0.8049, *p-*value = 0.9674 for *Ph. ariasi*, *Ph. perniciosus*, *Ph. mascittii* and *Se. minuta* captured by ST; and *p-*value = 0.9511, *p-*value = 0.9957, *p-*value = 0.3204, *p-*value = 0.7226 for *Ph. ariasi*, *Ph. perniciosus*, *Ph. mascittii* and *Se. minuta* captured with LT, respectively).

### Altitude distribution

**Fig. 4:**
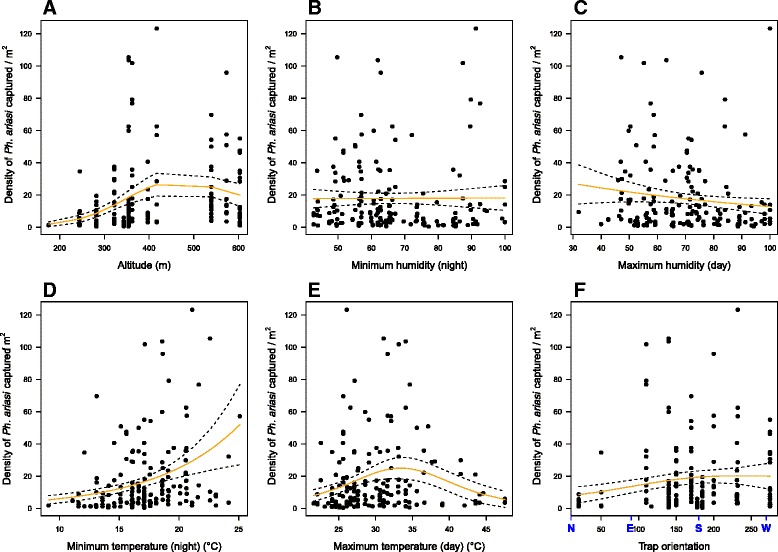
*Phlebotomus ariasi* density (sandflies/m^2^) relative to altitude (**a**), minimum relative humidity (**b**), maximum relative humidity (**c**), minimum temperature (**d**), maximum temperature (**e**) and trap orientation (in degree) with N = North, E = East, S = South and W = West (**f**). Black dots: field data; lines: selected Generalized Additive Models. Temperature and relative humidity were calculated during the monthly captures by separating the temperature/humidity data collected during the day and during the night

**Fig. 5:**
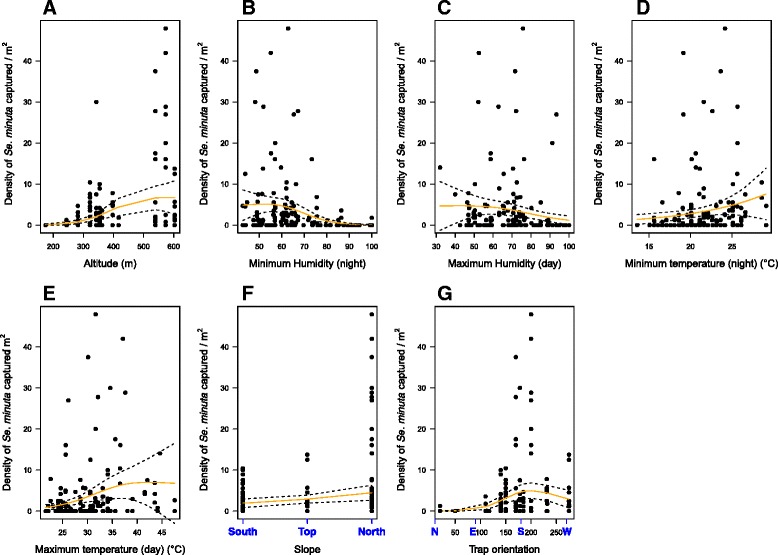
*Sergentomyia minuta* density (sandflies/m^2^) relative to altitude (**a**), maximum relative humidity (**b**), minimum relative humidity (**c**), minimum temperature (**d**), maximum temperature (**e**), slope (**f**) with South (100-500 m), Top (500-600 m) and North (200-500 m) and trap orientation (in degree) with N = North, E = East, S = South and W = West (**g**). Black dots: field data; lines: selected Generalized Additive Models. Temperature and relative humidity were calculated during monthly captures by separating the temperature/humidity data collected during the day and during the night. Only the graphics for significant results are shown

*Ph. ariasi* and *Se. minuta* were found at each altitude interval, with a peak of abundance at mid slope (300-400 m) for *Ph. ariasi* and at the summit (500-600 m) for *Se. minuta* (Table 4). A significant positive relationship was found between the presence (based on LT and ST captures) of *Ph. ariasi* (*p-*value < 0.001) and *Se. minuta* (*p-*value < 0.001) and the altitude (Fig. 3a and e). The abundance of these species, evaluated by ST captures, was also positively correlated with altitude (*p-*value < 0.0001 for both species). *Ph. ariasi* abundance increased until it reached a plateau between 400 m and 500 m and after 500 m started to decrease (Fig. 4a). *Se. minuta* abundance reached a plateau at 500 m (Fig. 5a).

**Table 4:** Density of sandfly species collected at the different altitude ranges during the three-year survey in Roquedur-le-Haut. Higher densities at mid slopes are observed

		Sticky Traps (density: sandflies/m^2^)			
Groups	Year	*Phlebotomus ariasi*	*Sergentomyia minuta*	*Phlebotomus perniciosus*	*Phlebotomus mascittii*
1. Hérault valley (100-300 m)	2011	3.32	0.00	0.09	0.00
	2012	1.44	0.00	0.00	0.00
	2013	2.85	0.14	0.07	0.06
2. Mid-slope/South side (300-400 m)	2011	40.11	1.24	0.92	0.17
	2012	15.95	2.10	0.58	0.05
	2013	24.04	3.20	0.09	0.06
3. Summit (500-600 m)	2011	26.25	7.79	0.33	0.00
	2012	19.41	6.29	0.09	0.00
	2013	35.46	9.61	0.00	0.00
4. Mid-slope/North side (300-500 m)	2011	38.84	0.90	0.00	0.00
	2012	25.75	1.69	0.00	0.00
	2013	50.43	1.04	0.15	0.00
5. Arre Valley (200-300 m)	2011	9.46	8.26	0.47	0.00
	2012	3.12	1.72	0.00	0.00
	2013	5.44	1.07	0.00	0.00
Light Traps (density: sandflies/night/trap)					
Groups	Year	*Ph. ariasi*	*Se. minuta*	*Ph. perniciosus*	*Ph. mascittii*
1. Hérault valley (100-300 m)	2011	0.15	0.00	0.00	0.00
	2012	0.13	0.00	0.00	0.00
	2013	0.36	0.00	0.00	0.00
2. Mid-slope/ South side (300-400 m)	2011	2.36	0.00	0.00	0.00
	2012	2.50	0.00	0.01	0.01
	2013	6.93	0.00	0.00	0.03
3. Summit (500-600 m)	2011	1.11	0.00	0.01	0.00
	2012	19.28	0.00	0.01	0.01
	2013	39.71	0.00	0.07	0.01
4. Mid-slope/North side (300-500 m)	2011	0.08	0.00	0.00	0.00
	2012	0.04	0.24	0.00	0.00
	2013	0.28	0.02	0.00	0.00
5. Arre Valley (200-300 m)	2011	3.82	0.00	0.07	0.00
	2012	0.94	0.00	0.00	0.01
	2013	4.81	0.00	0.03	0.00

### Effect of climatic conditions

Based on the analysis of the data collected by the temperature/humidity data loggers, no significant difference in temperature and relative humidity was observed between years (Kruskal-Wallis test, *p-*value = 0.9535 for temperature and *p-*value = 0.7413 for relative humidity) (Table 5). We found a positive relationship between the presence (LT and ST) of *Ph. ariasi* and *Se. minuta* and the mean temperature (*p-*value < 0.0001, for the two species) (Fig. 3b and f) and a significant negative relationship between the presence of *Ph. ariasi* and *Se. minuta* and the relative humidity (*p-*value < 0.0001, for both species) (Fig. 3c and g).

**Table 5:** Average/maximum/minimum temperature (°C) and relative humidity (%) during the capture period (May to September) for 2011, 2012 and 2013

	Temperature (°C)			Relative humidity (%)		
	Average	Maximum	Minimum	Average	Maximum	Minimum
2011	21.3	46.1	2.6	60.1	100	13.8
2012	20.4	46.6	5.6	70.6	100	20.9
2013	19.9	47.6	4.6	69.4	100	25.7

The abundance of *Ph. ariasi*, based on the ST captures, tended to be negatively correlated with the maximum relative humidity during the day (*p-*value = 0.0533) (Fig. 4c). On the other hand, *Se. minuta* abundance was negatively correlated with the minimum relative humidity during the night and the maximum relative humidity during the day (*p-*value < 0.0001 for minimum and *p-*value = 0.0172 for maximum relative humidity) (Fig. 5b and c).

We observed a positive relationship between the abundance (ST captures) of *Ph. ariasi* and *Se. minuta* and temperature. *Ph. ariasi* abundance increased with the temperature until 35 °C and above this temperature, it started to decrease (*p-*value < 0.0001 and *p-*value < 0.001 for minimum (night) and maximum (day) temperature, respectively) (Fig. 4d). *Se. minuta* abundance also increased with the temperature (*p-*value = 0.0292 and *p-*value = 0.00304 for minimum (night) and maximum (day) temperature respectively) (Fig. 5d and e).

### Effect of slope and wall orientation

In 1980, Rioux *et al.* suspected an effect of the slope to explain the altitude distribution of sandfly populations along this transect. For *Ph. ariasi* and *Se. minuta*, no significant correlation between their presence (ST and LT captures) and slope or wall orientation was found (*p-*value = 0.23124 and *p-*value = 0.0947 for slopes, *p-*value = 0.145668 and *p-*value = 0.7153 for wall orientation for *Ph. ariasi* and *Se. minuta,* respectively) (Fig. 3d and h).

However, a significant correlation between abundance (based on ST captures) of *Ph. ariasi* and wall orientation was found (*p-*value = 0.018) as well as a significant correlation between *Se. minuta* and slope (*p-*value = 0.0158) and wall orientation (*p-*value < 0.001) (Figs. 4f, 5f and g).

### Inter-annual relationship of temperature and *Ph. ariasi* abundance

**Fig. 6:**
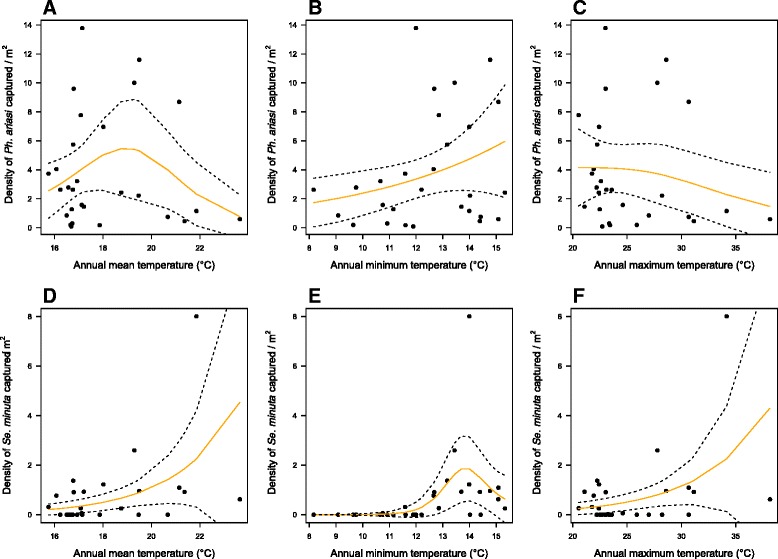
Relationship (Generalized Additive Models) between climatic variables and sandfly density (mean of the three years). *Phlebotomus ariasi* density (sandflies/m^2^) and annual mean temperature (**a**), annual minimum temperature (**b**) and annual maximum temperature (**c**): *Sergentomyia minuta* density (sandflies/m^2^) and annual mean temperature (**d**), annual minimum temperature (**e**) and annual maximum temperature (**f**). In comparison with *Ph. ariasi*, *Se. minuta* shows higher tolerance to higher temperature

The abundance of *Ph. ariasi* was related to the annual mean/minimum/maximum temperature (*p-*value < 0.0001 for all three) (Fig. 6a, b and c). *Ph. ariasi* abundance increased with the annual mean temperature until 20 °C and above this temperature it decreased. *Se. minuta* abundance also increased with the annual mean/minimum/maximum temperature, but without any limiting temperature (*p-*value = 0.00146, *p-*value = 0.0551 and *p-*value = 0.004, respectively) (Fig. 6d, e and f).

### Comparison with the data collected in 1977

In 1977, 5043 individuals (4215 males and 828 females) were captured by Rioux *et al*. (1980). Their abundance was comparable to that of the current study: *Ph. ariasi* (86.36 %), *Se. minuta* (13.27 %), *Ph. perniciosus* (0.26 %) and *Ph. mascittii* (0.12 %) (Kruskal-Wallis test, *p-*value = 0.9899).

When we compared the data from the 14 stations in common between the two studies (Table 1), the same peaks of density for *Ph. ariasi* (at mid slope) and *Se. minuta* (at the top) were observed (Table 6).

**Table 6:** Density of sandflies per m^2^ according to species collected in the 14 stations in common with the study of Rioux et al. [10] at the different altitude ranges in the studied area. Higher densities at mid slopes are observed

Sticky Traps Density: sandflies/m^2^ [Percentage (%)^a^]	Year	*Phlebotomus ariasi*	*Sergentomyia minuta*	*Phlebotomus perniciosus*	*Phlebotomus mascittii*
Group1. Hérault valley (100-300 m)	1977	5.12 [96.6 %]	0.00 [0.0 %]	0.18 [3.4 %]	0.00 [0.0 %]
	2011	3.32 [97.4 %]	0.00 [0.0 %]	0.09 [2.6 %]	0.00 [0.0 %]
	2012	1.44 [100 %]	0.00 [0.0 %]	0.00 [0.0 %]	0.00 [0.0 %]
	2013	2.85 [91.3 %]	0.14 [4.5 %]	0.07 [2.2 %]	0.06 [1.9 %]
Group2. Mid-slope / South side (300-400 m)	1977	29.84 [86.2 %]	4.18 [12.1 %]	0.36 [1.0 %]	0.22 [0.6 %]
	2011	38.50 [94.9 %]	1.24 [3.1 %]	0.78 [1.9 %]	0.04 [0.1 %]
	2012	13.93 [84.3 %]	2.10 [12.7 %]	0.45 [2.7 %]	0.05 [0.3 %]
	2013	23.29 [87.4 %]	3.20 [12.0 %]	0.09 [0.3 %]	0.06 [0.2 %]
Group3. Summit (500-600 m)	1977	27.59 [85.6 %]	4.66 [14.4 %]	0.00 [0.0 %]	0.00 [0.0 %]
	2011	26.25 [76.4 %]	7.79 [22.7 %]	0.33 [1.0 %]	0.00 [0.0 %]
	2012	17.11 [72.8 %]	6.29 [26.8 %]	0.09 [0.4 %]	0.00 [0.0 %]
	2013	32.70 [77.6 %]	9.42 [22.4 %]	0.00 [0.0 %]	0.00 [0.0 %]
Group4. Mid-slope / North side (300-500 m)	1977	63.65 [94.7 %]	3.54 [5.3 %]	0.00 [0.0 %]	0.00 [0.0 %]
	2011	34.52 [99.1 %]	0.30 [0.9 %]	0.00 [0.0 %]	0.00 [0.0 %]
	2012	24.29 [98.3 %]	0.43 [1.7 %]	0.00 [0.0 %]	0.00 [0.0 %]
	2013	44.14 [99.8 %]	0.00 [0.0 %]	0.08 [0.2 %]	0.00 [0.0 %]
Group5. Arre valley (200-300 m)	1977	19.18 [92.3 %]	1.40 [6.7 %]	0.00 [0.0 %]	0.20 [1.0 %]
	2011	7.16 [45.7 %]	8.26 [52.7 %]	0.25 [1.6 %]	0.00 [0.0 %]
	2012	2.99 [63.5 %]	1.72 [36.5 %]	0.00 [0.0 %]	0.00 [0.0 %]
	2013	5.21 [83.0 %]	1.07 [17.0 %]	0.00 [0.0 %]	0.00 [0.0 %]

^a^Relative abundance: nx/N * 100, nx: number of individuals belonging to species x, N: total number of sampled individuals

As no climatic record was available for 1977, we could not compare these data.

## Discussion

For this study, two different trap types were used (LT and ST) because they provide complementary information on the sandfly host-seeking and resting populations. LT preferentially target highly phototropic species [2], and allow capturing large numbers of samples and measuring the relative changes in abundance over time and space [25]. Moreover, LT are useful to determine the seasonal activity because they capture active sandflies. On the other hand, ST are not expensive, can be easily produced in large numbers and are a method of capture by interception rather than attraction. Therefore, they provide data on resting individuals [9]. Finally, some sandfly species, such as *Se. minuta* in Southern France, are not attracted by light traps.

In the present study, three *Phlebotomus* and one *Sergentomyia* species were identified in the Roquedur area, among which *Ph. ariasi* and *Ph. perniciosus*, the two major known vectors of leishmaniasis in France. Their spatiotemporal distribution (month/station) did not significantly change during the study period. Similar to previous studies [10, 14], *Ph. ariasi* was the predominant species (93.23 %) and *Se. minuta* was the second most abundant one in the study area (6.18 %). *Ph. perniciosus,* which accounted for only 0.48 % of captures in the study area, is fairly abundant in the Provence region [26]. In agreement with previous data [27], *Ph. mascittii* represented a very small proportion of the total sandfly captures (0.11 %). This low density may be due to a specific ecological niche for this species (such as caves or tunnels) [10, 28, 29].

Sandfly activity in the region started in May and ended in October, with a peak in July-August when the average temperature was optimal for sandflies (between 20 and 30 °C). *Ph. ariasi* and *Ph. perniciosus* were abundant in anthropic biotopes. Conversely, *Se. minuta* was more frequently captured in “semi-rural” stations. This difference could be possibly explained by their trophic preferences: mammals and avian species (especially domestic species such as dog, sheep and chicken) for *Ph. ariasi* and *Ph. perniciosus,* and reptiles for *Se. minuta* [2, 30].

Sandfly presence/abundance was significantly influenced by altitude. Nevertheless, *Ph. ariasi* was collected at almost all altitudes with a peak of abundance in mid-slope areas. Its highest densities were recorded between 400 and 500 m of altitude, although this species can be found >800 m above sea level and also in subalpine and mountain climates [9, 31]. *Se. minuta* was present at all altitudes with the highest total number at the summit (500–600 m). Similar results were also reported by Rioux et al. [10]. Although altitude is not an ecological factor by itself, sandfly distribution is influenced by environmental biotic and abiotic features related to altitude, such as temperature [32–34]. However, based on the data collected with LT, *Ph. ariasi* and *Ph. perniciosus* abundances seem to be also strongly influenced by the host abundance and availability, regardless of the altitude. For example, in the poultry farm located in station 10 (606 m), where many hosts were available, more than one thousand individuals were captured with a LT in one night. In contrast, *Se. minuta* prefers wild habitats with the highest abundance in “semi-rural” stations situated at the summit.

Besides altitude, sandfly presence and abundance were significantly influenced by temperature and relative humidity. Specifically, *Ph. ariasi* abundance increased with the temperature until 35 °C and decreased with the increasing of relative humidity. Similar findings were reported for *Ph. ariasi* in Spain [31]. This species can be found in a variety of habitats and usually prefers relatively high temperatures with moderate relative humidity. According to the literature, the lowest temperature of activity for *Ph. ariasi* is around 15 °C [35], its optimal nocturnal temperature ranges between 19 and 21 °C [9] and the maximum temperature is around 30 °C [36]. This is in agreement with our findings: lowest temperature of activity around 11-12 °C, optimal nocturnal temperature between 20 and 25 °C and maximum temperature around 35 °C. In comparison with *Ph. ariasi*, *Se. minuta* shows a higher tolerance to higher temperatures and lower relative humidity. In our study, the lowest temperature for this species was around 20 °C, its abundance increased with the temperature and decreased rapidly with a relative humidity higher than 60 %. No maximum temperature was detected. Moreover, our findings indicate that the effect of temperature on sandfly abundance is non-linear, and therefore, simplistic approaches about the influence of climate change on sandfly populations must be avoided. Indeed, higher temperatures may have a negative effect on sandfly populations, as described for other vectors, such as mosquitoes [37].

Previous studies suspected that slope and wall orientation may have a significant impact on the presence/abundance of sandflies [10, 38, 39]. In our study, *Ph. ariasi* and *Se. minuta* were more abundant in South-oriented slopes and South-oriented walls. From our knowledge, the impact of slope and wall orientation on sandfly presence and abundance has been very little studied, whereas these parameters appear to be important factors to consider in the framework of ecological studies. These two factors, which are also related to temperature and relative humidity, can be used as good predictors of sandfly abundance.

The comparison with the data from the study carried out in the same area in 1977 [10] allowed investigating the changes in the sandfly population during the last 30 years. The environment has considerably changed in 30 years. We observed an increase of human population and number of houses between 1982 and 2009 in Saint-Julien-de-la-Nef and Roquedur that may reflect a population movement from urban towards rural areas during this period (Table 2). The consequences of habitat transformation by urbanization, housing improvement and reduction of host abundance could also have affected the sandfly population. Moreover, Rioux et al. [14] reported that the annual mean temperature has increased over the years. However, data comparison did not highlight any significant difference in the relative abundance and spatiotemporal distribution of the different species between studies (2011 to 2013 *versus* 1977), despite the different methodologies: ST were left for 15 days in the study by Rioux et al. [10] and only for two nights in this study. This suggests that the sandfly population in this area is stable and, therefore, can adapt to environmental changes. Because of the methodological differences, the densities of sandflies by m^2^ could not be compared.

According to Ashford et al. [35], in Mediterranean regions, the temperature of leishmaniasis foci are generally between 20 °C and 30 °C in July and between 5 °C and 10 °C in January. Indeed, these temperatures are optimal for the development of large populations of *Ph. ariasi* and *Ph. perniciosus*, the two main leishmaniasis vectors in the South of France. However, no information about *Leishmania* transmission during the last 30 years is available. The increase of temperature and the impact of socio-ecological changes in this region could have modified *Leishmania* transmission. Environmental, population and individual factors have to be considered together to understand epidemiology and transmission ecology of parasites [40]. Rioux et al. [41] determined that in France, an area with a mean sandfly density (based on ST capture) above 20 sandflies/m^2^ is an area with high leishmaniasis transmission. Our study area (*Ph. ariasi* maximum density: 40.11, 25.75 and 50.43 specimens/m^2^ in 2011, 2012 and 2013, respectively) can be considered as a zone at risk of leishmaniasis transmission.

## Conclusions

This study provides useful information about the environmental and climatic factors, such as altitude, temperature, relative humidity, slopes and wall orientation, that can affect the presence and abundance of *Ph. ariasi* and *Se. minuta*. Our findings indicate that all these factors must be taken into account in a species-specific analysis of sandflies to evaluate the risk of *Leishmania* transmission. Although the environment has been considerably transformed in our study area in 30 years, sandfly abundance has not significantly changed between 1977 and 2011–2013, highlighting the sandfly capacity to adapt, in the short and long term, to the ecosystem modifications.

Our findings show that the study area, which is located in the Cévennes range of mountains with favorable topographic and climatic conditions for sandflies, has a rich sandfly population and harbors important vector species, such as *Ph. ariasi.* The high *Ph. ariasi* population density throughout the active season represents a risk of *L. infantum* transmission. Moreover, in the context of climate change, it is important to study the optimal conditions for vector species, the non-linear effects of temperature and to collect information on changes of these insect populations. Careful monitoring of environmental variables and their effect on vector biology is a key issue for the implementation of control strategies.
